# Inhibition of NF-kappaB transcriptional activity enhances fucoxanthinol-induced apoptosis in colorectal cancer cells

**DOI:** 10.1186/s41021-018-0116-1

**Published:** 2019-01-22

**Authors:** Shuya Tamura, Takumi Narita, Gen Fujii, Shingo Miyamoto, Takahiro Hamoya, Yurie Kurokawa, Maiko Takahashi, Kouhei Miki, Yui Matsuzawa, Masami Komiya, Masaru Terasaki, Tomohiro Yano, Michihiro Mutoh

**Affiliations:** 1 Epidemiology and Prevention Division, Research Center for Cancer Prevention and Screening, National Cancer Center, 5-1-1 Tsukiji, Chuo-ku, Tokyo, 104-0045 Japan; 2 Graduate School of Food and Nutritional Sciences, Toyo University, 1-1-1 Izumino, Itakura, Oura, Gunma, 374-0193 Japan; 3 Central Radioisotope Division, National Cancer Center Research Institute, 5-1-1 Tsukiji, Chuo-ku, Tokyo, 104-0045 Japan; 4 Department of Cancer Cell Research, Sasaki Institute, Sasaki Foundation, 2-2, Kandasurugadai Chiyoda-ku, Tokyo, 101-0062 Japan; 5 Division of Carcinogenesis and Cancer Prevention, National Cancer Center Research Institute, 5-1-1 Tsukiji, Chuo-ku, Tokyo, 104-0045 Japan; 6 School of Pharmaceutical Sciences, Health Sciences University of Hokkaido, 1757 Kanazawa, Ishikari-Tobetsu, Hokkaido 061-0293 Japan

**Keywords:** Colorectal cancer, Fucoxanthin, Fucoxanthinol, Apoptosis, NF-κB

## Abstract

**Background:**

Evidence from epidemiological and experimental studies has shown that the etiology of colorectal cancer (CRC) is related to lifestyle, mainly diet. At the same time, there are many foods and beverages that have been shown to provide protection against CRC. We turned our attention to a traditional Japanese food, brown algae, that contains carotenoids and various functional polyphenols, especially fucoxanthin (FX) and fucoxanthinol (FxOH).

**Results:**

Both FX and FxOH treatments induced apoptosis in a dose-dependent and time-dependent manner as detected by annexin V / propidium iodide and the presence of a subG1 population in human colon cancer HCT116 cells. This apoptotic effect of FxOH was stronger than that of FX. We also found that nuclear factor-kappa B (NF-κB) transcriptional activity was significantly increased by treatment with ≥5 μM FxOH. Thus, we cotreated the cells with FxOH plus NF-κB inhibitor, and the results demonstrated that this cotreatment strongly enhanced the induction of apoptosis compared with the effects of FxOH or NF-κB inhibitor treatment alone and resulted in X-linked inhibitor of apoptosis (IAP) downregulation.

**Conclusions:**

This study suggested that FxOH is a more potent apoptosis-inducing agent than FX and that its induction of apoptosis is enhanced by inhibiting NF-κB transcriptional activity via suppression of IAP family genes.

## Introduction

Colorectal cancer (CRC) is the third most common cancer in men (746,000 cases) and the second most common in women (614,000 cases) worldwide [1]. More than 50% of the cases occur in more developed countries [1], including Japan. Although there are decreasing trends in the rates of CRC incidence and mortality in highly developed countries, the rates are rising rapidly in many low- and middle-income countries [2]. In Japan, the National Cancer Research Center reported that CRC was the second most common cause of cancer death in 2016, and it is expected that the number of CRC patients will continue to increase [3]. Thus, establishment of preventive measures is strongly desired.

There is strong evidence that the etiology of CRC is related to lifestyle, mainly diet. Recently, the World Cancer Research Fund International Continuous Update Project, which provides a systematic review and meta-analysis of prospective studies to evaluate the dose-response risks between food and beverage intake and CRC, reported that high intake of red and processed meat and ethanol increase the risk of CRC [4]. At the same time, milk and whole grains may play a protective role against CRC. The evidence for vegetables and fish was less convincing [4]. There are many foods and beverages that have been demonstrated to play protective role against CRC, such as fruits, coffee and tea. However, there may be more foods that have not yet been identified as useful for cancer prevention. One food that we are interested in is brown algae. In addition to vitamins, minerals and dietary fiber, brown algae are known to contain many proteins, polysaccharides, carotenoids and various functional polyphenols [5].

Fucoxanthin (FX) is a xanthophyll belonging to the non-provitamin A carotenoids and is a unique carotenoid constructed with an unusual allenic bond, an epoxide group, and a conjugated carbonyl group in a polyene chain. When humans ingest FX, the acetyl group of FX is converted to a hydroxyl group by hydrolysis in the intestine epithelial cells, and it is metabolized to fucoxanthinol (FxOH) [5]. FX has been reported to reduce obesity, inflammation, triglyceride levels and to control high blood pressure in humans [6, 7]. We recently demonstrated that FxOH possesses anti-sphere formation capacities in CRC stem-like cells through its downregulation of integrin, mitogen-activated protein kinase (MAPK) and signal transducer and activator of transcription (Stat) signaling under normoxic and hypoxic conditions [8, 9]. Moreover, we reported that FxOH rapidly detached human CRC cells (DLD-1 cell line) from a culture dish and induced anoikis-like cell death through the suppression of integrin β1 and inactivation of focal adhesion kinase [10]. To date, anticancer activities of FX and FxOH have been reported, but the mechanism has not been fully elucidated.

In this study, we investigated the effects of FX and FxOH on the induction of apoptosis in CRC cells and found that combination treatment with nuclear factor-kappa B (NF-κB) inhibitor synergistically increased apoptosis induction.

## Methods

### Chemicals

FX was obtained from Cayman Chemical (Ann Arbor, MI, USA). FxOH was obtained from Wako Pure Chemical Industries Ltd. (Osaka, Japan) or was kindly supplied by Oryza Oil & Fat Chemical Co., Ltd. (Ichinomiya City, Aichi, Japan). SM-7368 was obtained from Cayman Chemical.

### Cell culture

HCT116 human colon adenocarcinoma cells were purchased from the American Type Culture Collection (Manassas, VA, USA). HCT116 cells were maintained in DMEM supplemented with 10% heat-inactivated fetal bovine serum (FBS; HyClone Laboratories Inc., Logan, UT, USA) and antibiotics (100 μg/ml streptomycin and 100 U/ml penicillin) at 37 °C with 5% CO_2_.

### Apoptosis assay

Cells were plated in 24-well plates (5 × 10^4^ cells/well) and treated with FX (25 μM), FxOH (1, 5 or 10 μM), SM-7368 (20 μM) and in combination. Cells were harvested 12, 24, 36 and 48 h posttreatment and stained using annexin-V Alexa Fluor® 488/PI (propidium iodide), as described by the Tali® Apoptosis Kit – Annexin V Alexa Fluor® 488 and propidium iodide (Life Technologies Corporation, Van Allen Way Carlsbad, CA, USA). Cell viability, death and apoptosis were evaluated using the Tali® Image-based Cytometer (Life Technologies Corporation). The annexin-V positive/PI-negative cells were recognized as early apoptotic cells by the cytometer, whereas the annexin V-positive/PI-positive cells were identified as late apoptotic cells. Similarly, the annexin V-negative/PI-negative cells were identified as viable cells and the annexin V-negative/PI-positive cells were identified as dead cells.

### Cell cycle assay

Cells were plated in 24-well plates (5 × 10^4^ cells/well) and treated with FX (25 μM), FxOH (1, 5 or 10 μM), SM-7368 (20 μM) and in combination. Cells were harvested 24, 36 and 48 h posttreatment and stained using PI, as described by the Tali® Cell Cycle Kit (Life Technologies Corporation). Cell cycle distributions were evaluated using the Tali® Image-based Cytometer (Life Technologies Corporation).

### Luciferase assays for NF-κB promoter transcriptional activity

HCT116 human colon cancer cells were transfected with the pNL1.3 [secNluc/NF-κB-RE/Puro] reporter plasmid using polyethylenimine MAX MW 40,000 (PolyScience, Warrington, PA, USA). Transfectants stably expressing NF-κB-secNluc were obtained after treatment with puromycin and cloning for use in measuring NF-κB transcriptional activity. The cells were referred to as HCT116-NF-κB-secNluc cells. HCT116-NF-κB-secNluc cells were seeded in 96-well plates (2 × 10^4^ cells/well). After 24 h incubation, the cells were treated with 1, 5 or 10 μM fucoxanthinol for 24 h. Firefly luciferase activity levels were determined using the Nano-Glo® Luciferase Assay System (Promega Corporation). The basal NF-κB luciferase activity in the control was set as 1.0. Data are expressed as the means ± SD (*n* = 6).

### Protein extraction and western blotting

HCT116 cells were lysed with RIPA (radioimmunoprecipitation assay) buffer (50 mM Tris-HCl, pH 8.0, 150 mM NaCl, 1% Triton X-100, 0.5% deoxycholate and 0.2% SDS) and sonicated. Protein concentrations in the soluble fractions were determined using the DC™ Protein Assay (Bio-Rad Laboratories, Hercules, CA, USA). The extracted protein samples were standardized by adding RIPA buffer, 2-mercaptoethanol and bromophenol blue to obtain final concentrations of 5 and 0.05%, for the latter two compounds, respectively, before the samples were boiled for 5 min. Fifteen micrograms of protein from each sample was subjected to SDS-PAGE followed by transfer to polyvinylidenedifluoride membranes (Merck, Darmstadt, Germany). Membranes were blocked with 2.5% FBS in Tris-buffered saline (50 mM Tris, pH 7.4, and 150 mM NaCl) containing 0.1% Tween 20 (TBS-T) for 1 h at room temperature and then probed with appropriate primary antibodies overnight at 4 °C. Primary antibodies for PARP (Cell Signaling, Boston, MA), cIAP-1 (Cell Signaling), XIAP (Cell Signaling), cleaved Caspase3 (Cell Signaling), and β-actin (Merck) were used. After several washes with TBS-T, membranes were incubated with an appropriate secondary antibody (ECL™ anti-mouse or anti-rabbit IgG, horseradish peroxidase-linked F(ab’)2 fragment (from sheep); GE Healthcare, Buckinghamshire, UK) for 1 h at room temperature. After washing with TBS-T, membranes were incubated with ECL western blotting detection reagents (GE Healthcare, Buckinghamshire, UK). Specific proteins were visualized with a LAS4000 system (GE Healthcare), and the data were analyzed using Multigauge software (Fujifilm).

## Results

### Fucoxanthinol strongly induced cell death compared with fucoxanthin

**Fig. 1:**
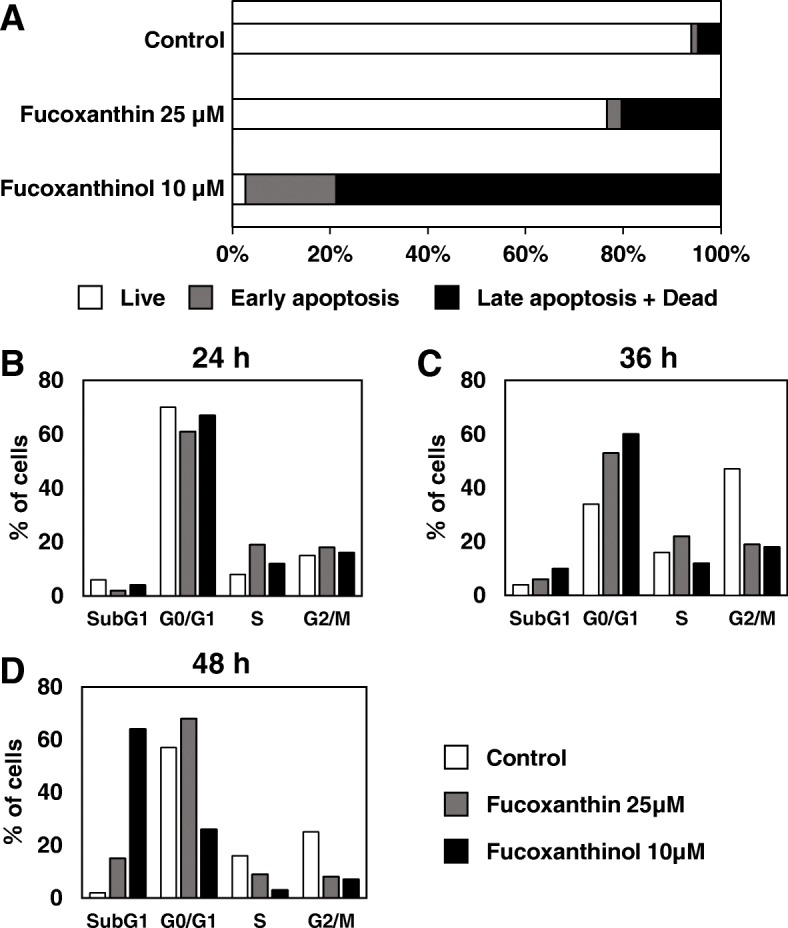
Fucoxanthinol strongly induced cell death compared with fucoxanthin. **a** HCT116 cells were treated with fucoxanthin (25 μM) or fucoxanthinol (10 μM) for 48 h. HCT116 cells then were stained with annexin V and propidium iodide (PI) to assess apoptosis. This apoptosis assay simultaneously provides quantitative data about the percentages of vital cells (annexin V (−) / PI (−)), early apoptotic cells (annexin V (+) / PI (−)), late apoptotic cells (annexin V (+) / PI (+)) and dead cells (annexin V (−) / PI (+)). **b**-**d** HCT116 cells were treated with fucoxanthin (25 μM) or fucoxanthinol (10 μM) for 24 (B), 36 (**c**) and 48 h (**d**). For the cell cycle assay, HCT116 cells were then stained with propidium iodide

To compare the effects of FX and FxOH on apoptosis induction, we performed apoptosis assays using annexin V and propidium iodide (PI) in HCT116 human colon cancer cells. Both FX and FxOH induced apoptotic cells, but the apoptosis effect of FxOH was stronger than that of FX (Fig. 1a). Furthermore, the fractions of late apoptotic cell plus dead cells were increased 13% or 52% by 25 μM FX or 10 μM FxOH treatment for 48 h, respectively, compared to the nontreated control cells (Fig. 1a).

In a previous report, a subG_1_ population has been shown to represent an apoptotic population due to the loss of fragmented DNA [11, 12]. Thus, we performed cell cycle assays to confirm apoptosis. A subG_1_ population was not clearly induced by treatment with 25 μM FX or 10 μM FxOH for 24 h. A small increase in the SubG_1_ population was observed 36 h after 10 μM FxOH treatment (Fig. 1c). Treatment with 10 μM FxOH for 48 h increased the SubG_1_ population to 62% (Fig. 1d). Strong induction of a SubG_1_ population was obtained by FxOH treatment for 48 h, and this result was similar to the data obtained in the annexin V assays (Fig. 1b-d). After this experiment, we focused our efforts on revealing the mechanism of FxOH-induced apoptosis.

### Fucoxanthinol induced apoptosis in a dose- and time-dependent manner

**Fig. 2:**
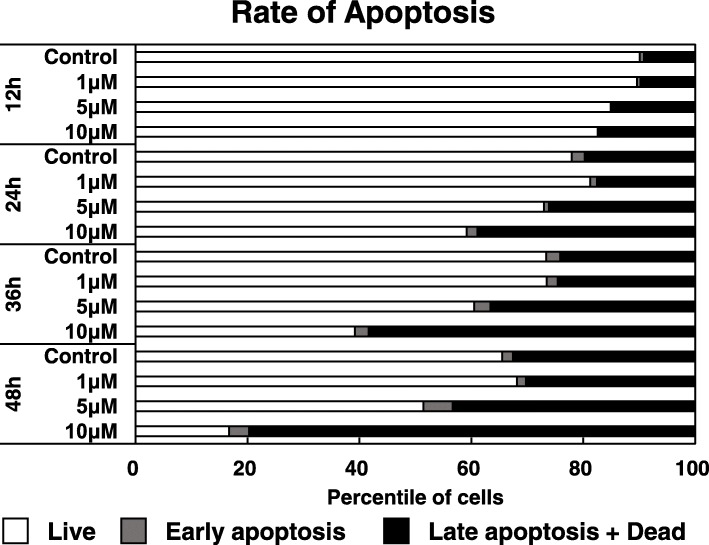
Fucoxanthinol-induced apoptosis depended on the concentration and treatment time. HCT116 cells were treated with fucoxanthinol (0, 5 and 10 μM) for 12, 24, 36 and 48 h. HCT116 cells then were stained with annexin V and propidium iodide (PI) to assess apoptosis. This apoptosis assay simultaneously provides quantitative data about the percentages of vital cells (annexin V (−) / PI (−)), early apoptotic cells (annexin V (+) / PI (−)), late apoptotic cells (annexin V (+) / PI (+)) and dead cells (annexin V (−) / PI (+))

We confirmed dose- and time-dependent induction of apoptosis by FxOH up to 48 h in HCT116 cells (Fig. 2). Early apoptosis was obvious at 24 h after regardless of any treatment, but the majority were live cells. Late apoptotic plus dead cells represented 80% of the population after 48 h treatment with 10 μM FxOH (Fig. 2).

### Enhancement of fucoxanthinol-induced apoptosis via inhibition of NF-κB

**Fig. 3:**
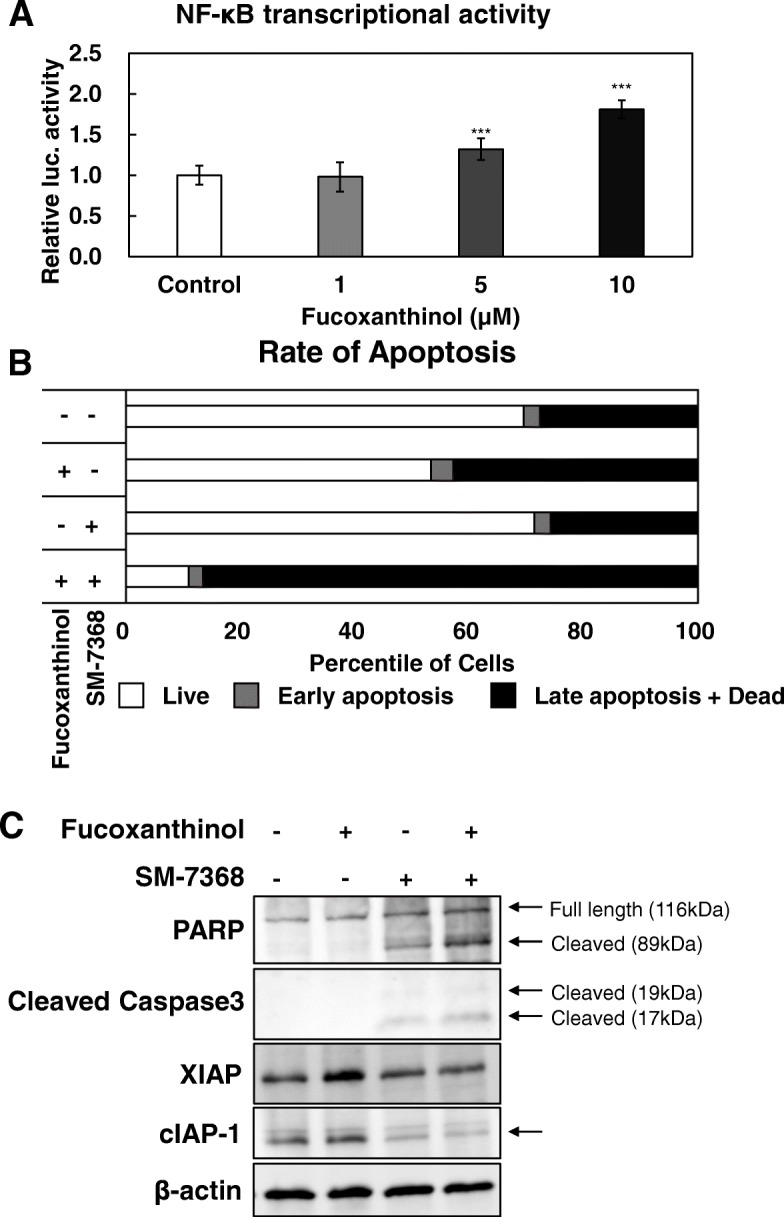
Fucoxanthinol-induced apoptosis was enhanced by NF-κB inhibitor. **a** HCT116_NF-κB_secNluc cells were treated with fucoxanthinol (0, 1, 5 and 10 μM) for 24 h. We then measured NF-κB promoter transcriptional activity in HCT116_NF-κB_secNluc cells using a reporter assay. The values shown in panel are the means ± S.D. *p < 0.05, **p < 0.01, ***p < 0.001 vs. control as analyzed by Student’s t-test after one-way ANOVA. **b** HCT116 cells were treated with fucoxanthinol (5 μM) and/or SM-7368 (20 μM for 36 h. HCT116 cells then were stained with annexin V and propidium iodide (PI) to assess apoptosis. Apoptosis assay simultaneously provides quantitative data about the percentages of vital cells (annexin V (−) / PI (−)), early apoptotic cells (annexin V (+) / PI (−)), late apoptotic cells (annexin V (+) / PI (+)) and dead cells (annexin V (−) / PI (+)). The data were gathered three time and representative data are shown. **c** HCT116 cells were treated with fucoxanthinol (5 μM) and/or SM-7368 (20 μM) for 36 h. This example immunoblot image shows the expression levels of apoptosis-related proteins and proteins downstream of NF-κB-downstream in HCT116 cells. β-actin was used as the internal control

It has been reported that the activation of the transcription factor NF-κB leads an antiapoptotic response through the upregulation of cell death repressors [13]. Thus, we first evaluated the effects of FxOH on NF-κB transcriptional activity in HCT116 cells. Contrary to our expectation, NF-κB transcriptional activity was significantly increased by treatment with 5 μM and 10 μM FxOH (Fig. 3a).

In many solid cancers, the constitutive activation of NF-κB has an important role on chemoresistance [14]. Thus, we hypothesized that the rather weak induction of apoptosis by FxOH may explained by activation of NF-κB and that inhibition of NF-κB transcriptional activity may enhance FxOH-induced apoptosis. In the next experiment, HCT116 cells were cotreated with FxOH plus NF-κB inhibitor to compare the apoptosis induction ability of the combination with that of FxOH alone. We used 5 μM of FxOH, as this dose enhanced NF-κB transcriptional activity. A 20 μM SM-7368 dose did not induce apoptosis. As expected, cotreatment of FxOH and SM-7368 strongly induced apoptosis compared with treatments with either FxOH or SM-7368 alone (Fig. 3b).

Finally, we analyzed the expression levels of apoptosis-related and NF-κB-downstream proteins. Treatment with FxOH upregulated the expression of downstream targets of NF-κB, including X-linked inhibitor of apoptosis protein (XIAP) and cellular inhibitor of apoptosis protein-1 (cIAP-1), whereas cotreatment of SM-7368 and FxOH inhibited the expression of downstream targets of NF-κB, similar to treatment with SM-7368 alone (Fig. 3c). It was shown that the expression levels of cleaved poly-(ADP-ribose) polymerase (PARP) and caspase-3 were increased after treatment with SM-7368, and the effect was even stronger after cotreatment with FxOH and SM-7368 (Fig. 3c).

## Discussion

In the present study, FxOH (rather than FX) was shown to be a functional food component that can induce apoptosis. It was newly established that FxOH may have some potential to enhance NF-κB transcriptional activity and that the inhibition of NF-κB transcriptional activity resulted in synergistic induction of apoptosis when combined with FxOH in human CRC cells.

FX and FxOH are known to be functional food components that have various activities, including an antiobesity effect. Moreover, it has been reported that FxOH could suppress tumor necrosis factor-α (TNF-α), and monocyte chemotactic protein-1 (MCP-1) mRNA expression and protein levels in a co-culture of adipocyte and macrophage cells [15]. This report suggested that FxOH may affect NF-κB target genes, regarding inflammatory and immune response, which are important for discovering novel drug. Unfortunately, evidence for anticancer effects of FX or FxOH is limited. As far as we know, clinical trials or clinical applications have not yet been performed. Regarding molecular mechanisms of Fx-induced apoptosis, it has been suggested that it is mediated via mitochondrial membrane permeabilization and caspase-3 activation [16]. FxOH-induced apoptosis is mediated via Bcl-2 inhibition [17]. In addition, we recently reported that FxOH rapidly detached DLD-1 cells from a dish culture and induced anoikis-like cell death with caspase-3 activation followed by a decrease of integrin β1 expression, and inhibition of focal adhesion kinase and Paxillin activation [10]. We suggested that the rather weak induction of apoptosis by FxOH in CRC cells may be partly explained by a concurrent increase in NF-κB transcriptional activity, which confers an antiapoptotic phenotype through IAP family induction. Thus, we conducted the next experiments to induce apoptosis using an NF-κB inhibitor in combination with FxOH. Interestingly, synergistic induction of apoptosis was observed rather than additive induction; therefore, these results indicate that apoptosis induction is greatly enhanced compared to single treatment with either FxOH or NF-κB inhibitor.

The IAP family proteins are responsible for limiting apoptosis, and they are regulated by activation of NF-κB [18]. It has been shown that the IAP family acts through direct and indirect inhibition of caspases [18]. For instance, XIAP directly inhibits pro-caspase 9 activation. XIAP, cAP1/2 and survivin inhibit the proteolytic function of caspases-3 and 7. The major IAP family proteins that are expressed in CRC are survivin, livin, XIAP, cIAP2 and NAIP [18]. Thus, IAPs are considered promising therapeutic targets for cancer treatment, and IAP-targeted therapy is now under clinical evaluation [18].

In this study, we conclude that FxOH itself can induce IAP-family proteins, and induction of IAP-family proteins do not provide a biological defense against FxOH-induced apoptosis. Our western blot experiments showed that the expression levels of IAP-family protein were increased by FxOH treatment alone under without the formation of cleaved PARP.

On the other hand, the expression level of XIAP was decreased by the FxOH plus NF-κB inhibitor combination (which led to a strong induction of apoptosis). These data support our hypothesis that the enhancement of apoptosis induction was caused by blocking antiapoptotic molecules. Of note, single treatments with FxOH or NF-κB inhibitor at the concentrations used in this study do not show strong cytotoxicity. In the data of Fig. 3c, PARP and caspase-3 were cleaved after treatment with only SM-7368. This means that SM-7368 induces apoptosis. On the other hand, FxOH treatment only did not induce PARP and caspase degradation. Thus, the data in Fig. 3c seem inconsistent with the data in Fig. 3b. It has been reported that the IAP family inhibits cleavage from pro-caspase-3 to caspase-3 [19]. Thus, NF-κB activation by FxOH is expected to cause the inhibition of cleaved caspase-3 protein expression via the activation of the IAP family. Therefore, treatment of FxOH may inhibit cleaved caspase-3 and cleaved PARP expression via NF-κB transcriptional activation. Meanwhile, treatment with NF-κB inhibitor, SM-7368, can induce cleaved caspase-3 and cleaved PARP expression. Thus, our findings are consistent with a previous paper.

The advantage of using an NF-κB inhibitor may be that it could lower the concentration of FxOH required to induce apoptosis, as there is a limit to how much brown algae can be consumed as a daily food. For example, the amount of FX contained in brown algae is 5–21 mg / 100 g (fresh Makonbu; *Laminaria japonica Areschoug*) [20]. Moreover, we could only obtain very low blood concentrations of FxOH via intake of brown algae. Previous examination suggested that a maximum serum concentration of FxOH is 44.2 nmol/L after administration of a single dose of 31 mg FX in adult human [20]. Thus, it is very difficult to obtain the pharmacological doses that we used in this study by food intake alone. Moreover, high doses of iodine are harmful for humans. The amount of iodine intake from seaweed has been estimated as 13.5–45 mg/day in the Japanese population [21]. Of note, the safe upper limit of iodine intake is 3 mg/day, which was set by the Ministry of Health, Labor and Welfare in Japan [22]. Thus, the development of concentrated FX or FxOH supplements is a realistic strategy for towards the development of cancer chemopreventive methods using FX or FxOH.

On the other hand, strong cytotoxicity is known for NF-κB inhibitors, and NF-κB is an important transcription factor for maintaining body homeostasis; therefore, it should be carefully used. Further research results are expected in the identification of food ingredients having inhibitory activities against NF-κB, such as epigallocatechin-3-gallate, caffeic acid phenethyl ester, and dried citrus peel [23–25].

This study targeted an intestinal tract cancer. Because dietary FX is converted to the deacetylated form FxOH by intestinal esterases, the FxOH concentration in the intestinal mucosae is expected to be higher than that in the blood. Thus, we speculated that anticancer effects are more likely to be obtained against intestinal cancer than against other organ cancers.

## Conclusions

This study suggested that FxOH is a more potent apoptotic inducer than FX. Induction of apoptosis by FxOH was enhanced using a NF-κB inhibitor via suppression of IAP family genes. When FxOH is used in combination with food components having an inhibitory action against NF-κB, effective anticancer effects or cancer preventative effects may be obtained with weak side effects. Further studies, such as in vivo and human clinical intervention studies, are required to conclusively establish FxOH as a promising prophylactic agent against CRC.

## Abbreviations

cIAP-1: Cellular inhibitor of apoptosis protein-1
CRC: Colorectal cancer
FBS: Fetal bovine serum
FX: Fucoxanthin
FxOH: Fucoxanthinol
IAP: Inhibitor of apoptosis
MAPK: Mitogen-activated protein kinase
NF-κB: Nuclear factor-kappaB
PARP: Poly-(ADP-ribose) polymerase
PI: Propidium iodide
RIPA: Radioimmunoprecipitation assay
Stat: Signal transducer and activator of transcription
XIAP: X-linked inhibitor of apoptosis protein

## References

[1] American Cancer Society. Cancer Facts and Figures 2015*.* Atlanta: American CancerSociety2015; Available from: https://www.cancerorg/content/dam/cancer-org/research/cancer-facts-and-statistics/annual-cancer-facts-and-figures/2015/cancer-facts-and-figures-2015.pdf.

[2] Arnold M, Sierra MS, Laversanne M, Soerjomataram I, Jemal A, Bray F (2017). Global patterns and trends in colorectal cancer incidence and mortality. Gut.

[3] Cancer Statistics in Japan '17. Center for Cancer Control and Information Services, National Cancer Center. Available from: https://ganjoho.jp/en/professional/statistics/brochure/2017_en.html

[4] Vieira AR, Abar L, Chan DSM, Vingeliene S, Polemiti E, Stevens C, Greenwood D, Norat T (2017). Foods and beverages and colorectal cancer risk: a systematic review and meta-analysis of cohort studies, an update of the evidence of the WCRF-AICR continuous update project. Ann Oncol.

[5] Terasaki M, Mutoh M, Fujii G, Takahashi M, Ishigamori R, Masuda S (2014). Potential ability of xanthophylls to prevent obesity-associated cancer. World J Pharmacol.

[6] Maeda H, Hosokawa M, Sashima T, Funayama K, Miyashita K (2007). Effect of medium-chain triacylglycerols on anti-obesity effect of fucoxanthin. J Oleo Sci.

[7] Abidov M, Ramazanov Z, Seifulla R, Grachev S (2010). The effects of Xanthigen in the weight management of obese premenopausal women with non-alcoholic fatty liver disease and normal liver fat. Diabetes Obes Metab.

[8] Terasaki M, Mima M, Kudoh S, Endo T, Maeda H, Hamada J, Osada K, Miyashita K, Mutoh M (2018). Glycine and succinic acid are effective indicators of the suppression of epithelial-mesenchymal transition by fucoxanthinol in colorectal cancer stem-like cells. Oncol Rep.

[9] Terasaki M, Ogawa Y, Endo T, Maeda H, Hamada J, Osada K, Miyashita K, Mutoh M (2018). Glycine is a predictor for a suppressive effect of fucoxanthinol on colonosphere formation under hypoxia. Anticancer Res.

[10] Terasaki M, Maeda H, Miyashita K, Mutoh M (2017). Induction of anoikis in human colorectal cancer cells by fucoxanthinol. Nutr Cancer.

[11] Darzynkiewicz Z, Bruno S, Del Bino G, Gorczyca W, Hotz MA, Lassota P, Traganos F (1992). Features of apoptotic cells measured by flow cytometry. Cytometry.

[12] Nicoletti I, Migliorati G, Pagliacci MC, Grignani F, Riccardi C (1991). A rapid and simple method for measuring thymocyte apoptosis by propidium iodide staining and flow cytometry. J Immunol Methods.

[13] Luo JL, Kamata H, Karin M (2005). The anti-death machinery in IKK/NF-kappaB signaling. J Clin Immunol.

[14] Wang CY, Cusack JC, Liu R, Baldwin AS (1999). Control of inducible chemoresistance: enhanced anti-tumor therapy through increased apoptosis by inhibition of NF-κB. Nat Med.

[15] Maeda H, Kanno S, Kodate M, Hosokawa M, Miyashita K (2015). Fucoxanthinol, metabolite of fucoxanthin, improves obesity-induced inflammation in adipocyte cells. Mar Drugs.

[16] Kotake-Nara E, Terasaki M, Nagao A (2005). Characterization of apoptosis induced by fucoxanthin in human promyelocytic leukemia cells. Biosci Biotechnol Biochem.

[17] Hosokawa M, Kudo M, Maeda H, Kohno H, Tanaka T, Miyashita K (2004). Fucoxanthin induces apoptosis and enhances the antiproliferative effect of the PPARgamma ligand, troglitazone, on colon cancer cells. Biochim Biophys Acta.

[18] Mohamed MS, Bishr MK, Almutairi FM, Ali AG (2017). Inhibitors of apoptosis: clinical implications in cancer. Apoptosis.

[19] Riedl SJ, Shi Y (2004). Molecular mechanisms of caspase regulation during apoptosis. Nat Rev Mol Cell Biol.

[20] Kanazawa K. High bioavailability and diverse biofunctions of Fucoxanthin in Brown algae. Nippon Shokuhin Kagaku Kogaku Kaishi 2012;59:49–55. (in Japanese).

[21] Zava TT, Zava DT (2011). Assessment of Japanese iodine intake based on seaweed consumption in Japan: a literature-based analysis. Thyroid Res.

[22] The Ministry of Health, Labour, and Welfare, Japan. In the Ministry of Health, Labour, and Welfare.Daiichi Shuppan Publishing. Tokyo; 2005. Dietary reference intakes for Japanese; pp. 189–193.

[23] Lin YL, Lin JK (1997). Epigallocatechin-3-gallate blocks the induction of nitric oxide synthase by down-regulating lipopolysaccharide-induced activity of transcription factor nuclear factor-kappaB. Mol Pharmacol.

[24] Natarajan K, Singh S, Burke TR, Grunberger D, Aggarwal BB (1996). Caffeic acid phenethyl ester is a potent and specific inhibitor of activation of nuclear transcription factor NF-kappa B. Proc Natl Acad Sci U S A.

[25] Onuma W, Asai D, Tomono S, Miyamoto S, Fujii G, Hamoya T, Nagano A, Takahashi S, Masumori S, Miyoshi N, Wakabayashi K, Mutoh M (2017). Anticarcinogenic effects of dried citrus peel in colon carcinogenesis due to inhibition of oxidative stress. Nutr Cancer.

